# A novel Family Dignity Intervention (FDI) for enhancing and informing holistic palliative care in Asia: study protocol for a randomized controlled trial

**DOI:** 10.1186/s13063-017-2325-5

**Published:** 2017-12-04

**Authors:** Andy Hau Yan Ho, Josip Car, Moon-Ho Ringo Ho, Geraldine Tan-Ho, Ping Ying Choo, Paul Victor Patinadan, Poh Heng Chong, Wah Ying Ong, Gilbert Fan, Yee Pin Tan, Robert A. Neimeyer, Harvey M. Chochinov

**Affiliations:** 1 Psychology, School of Social Sciences, Nanyang Technological University, 14 Nanyang Drive, HSS-04-03, Singapore, 637332 Singapore; 2 Centre for Population Health Sciences, Lee Kong Chian School of Medicine, Nanyang Technological University, 11 Mandalay Road, Level 18, Clinical Science Building, Singapore, 308232 Singapore; 3 HCA Hospice Care, 12 Jalan Tan Tock Seng, Singapore, 308437 Singapore; 4 Dover Park Hospice, 10 Jalan Tan Tock Seng, Singapore, 308436 Singapore; 5 Department of Psychosocial Oncology and Patient Support, National Cancer Centre Singapore, 11 Hospital Drive, Singapore, 169610 Singapore; 6 Department of Psychology, University of Memphis, Room 347, 202 Psychology Building, Memphis, TN 38152 USA; 7 Manitoba Palliative Care Research Unit, CancerCare Manitoba, 3017-675 McDermot Ave, Winnipeg, MB R3E 0V9 Canada

**Keywords:** Dignity, Family, Palliative care, End-of-life, Psycho-socio-spiritual intervention, Randomized controlled trial, Asia

## Abstract

**Background:**

The lack of a holistic approach to palliative care can lead to a fractured sense of dignity at the end of life, resulting in depression, hopelessness, feelings of being a burden to others, and the loss of the will to live among terminally ill patients. Building on the clinical foundation of Dignity Therapy, together with the empirical understanding of dignity-related concerns of Asian families facing terminal illness, a novel Family Dignity Intervention (FDI) has been developed for Asian palliative care. FDI comprises a recorded interview with a patient and their primary family caregiver, which is transcribed, edited into a legacy document, and returned to the dyads for sharing with the rest of the patient’s family. The aims of this study are to assess the feasibility, acceptability and potential effectiveness of FDI in reducing psychosocial, emotional, spiritual, and psychophysiological distress in community-dwelling and in-patient, Asian, older terminally ill patients and their families living in Singapore.

**Methods/design:**

An open-label randomized controlled trial. One hundred and twenty-six patient-family dyads are randomly allocated to one of two groups: (1) an intervention group (FDI offered in addition to standard psychological care) and (2) a control group (standard psychological care). Both quantitative and qualitative outcomes are assessed in face-to-face interviews at baseline, 3 days and 2 weeks after intervention, as well as during an exit interview with family caregivers at 2 months post bereavement. Primary outcome measures include sense of dignity for patients and psychological distress for caregivers. Secondary outcomes include meaning in life, quality of life, spirituality, hopefulness, perceived support, and psychophysiological wellbeing, as well as bereavement outcomes for caregivers. Qualitative data are analyzed using the Framework method.

**Discussion:**

To date, there is no available palliative care intervention for dignity enhancement in Asia. This first-of-its-kind study develops and tests an evidence-based, family driven, psycho-socio-spiritual intervention for enhancing dignity and wellbeing among Asian patients and families facing mortality. It addresses a critical gap in the provision of holistic palliative care. The expected outcomes will contribute to advancements in both theories and practices of palliative care for Singapore and its neighboring regions while serving to inform similar developments in other Asian communities.

**Trial registration:**

ClinicalTrials.gov, ID: NCT03200730. Registered on 26 June 2017.

**Electronic supplementary material:**

The online version of this article (doi:10.1186/s13063-017-2325-5) contains supplementary material, which is available to authorized users.

## Background

The defining principles of palliative care, including symptom control as well as psychological and spiritual support, aim to optimize quality of life and promote death with dignity [1]. However, most conventional palliative care interventions are heavily medically oriented, focusing predominantly on symptom management and control of physical pain, without addressing the psychosocial, emotional, and spiritual pains of the dying. The lack of a holistic approach to palliative care can lead to a fractured sense of dignity at the end of life. According to a vast body of academic literature conducted in the West, an undermining of dignity among dying patients is associated with high levels of depression, anxiety, hopelessness, feelings of being a burden to others, as well as the loss of the will to live [2, 3]. Conversely, patients deem a sense of control, hope, pride, self-respect, and self-esteem to be among the most important facets of facing death with dignity [4, 5]. An integrative review of the empirical research that examined the meaning of dying with dignity by Guo and Jacelon [6] supports these findings, and echoes Erickson’s theory of psychosocial development which highlights the need for elders to reflect on their lives with a sense of closure, completeness, and acceptance so as to experience ego integrity rather than despair at the last stage of life [7]. Clearly, palliative intervention must reach beyond the realm of physical care to fully address the total pain of mortality [8]. Yet, aside from the important work of religious clergies and faith-based counselors, the World Health Organization, together with numerous international bodies like the Worldwide Palliative Care Alliance and the Marie Curie Foundation, have repeatedly noted a general lack of non-pharmacological, structured psycho-social-spiritual interventions specifically designed to lessen the suffering and existential distress that accompany death and dying [9, 10].

Via interviews with older hospice patients in Canada, Chochinov and his colleagues [11, 12] developed an empirical model of dignity that identifies three major categories of physical, psychosocial and existential issues pertaining to one’s experience of dignity at life’s end. This model further forms the foundation of a brief psychotherapy to help reduce distress and promote dignity [13]. Specifically, dignity therapy involves a semi-structured interview with individual patients using nine core questions that stem from the dignity model. Patients are invited to respond to those questions that resonate as meaningful to them, and this often includes offering final words and remembrances to their loved ones. The interview is conducted by a trained therapist, which is recorded, transcribed, and edited. A follow-up session is held shortly after for the patient to review the edited transcript and to make conclusive modifications if required. Upon final revision, the patient is given a hard copy of the “generativity” document, and asked to identify with whom the document should be shared or bequeathed following death. A series of evaluation studies on Dignity Therapy conducted with hospice patients in Canada, the United States, and Australia generated positive findings for both patients and their families in elevating one’s sense of dignity, heightening life’s meaning, increasing the will to live, and providing comfort through grief [14, 15].

Both the dignity model and dignity therapy are well received and profoundly influential in the development of palliative care practices and policies in Western societies. However, the meaning of dignity varies across cultures and different ethnic groups due to its value-laden nature. Recognizing the critical role that families plays in rendering end-of-life care in Eastern societies as well as the longstanding emphasis of collectivism over individualism in Asia, Ho and his colleagues conducted an extensive body of research investigating the construct of dignity from the perspectives of older, Asian terminally ill patients and their family caregivers [16, 17]. While the findings from these investigations mostly supported the Western dignity model and its three core conceptual categories related to the physical, psychosocial, and existential domains, family relationships and family processes have emerged as critical components of dignity in the Asian model. Particularly, it was reported that older Asian patients experience great distress as they feel trapped in the liminal space between living and dying, challenged by limited mobility and increased dependency. According to Ho et al. [18], in order to maintain and promote dignity at life’s final margin, one must strengthen patients’ *spiritual plasticity* through helping them find meaning in their pain, let go of their attachments, and establish moral transcendence with the love and support of their families; equally important is to help patients bolster their sense of *family connectedness* through creating a platform for expressing appreciation, achieving reconciliation, fulfilling family obligations, and establishing continuing bonds with their descendants. Findings with family caregivers further revealed that patients’ sense of dignity can be enhanced through strengthening *family integrity* and *filial compassion*, which involves the cultivation of mutual support, reciprocal relationship, and altruistic reverence gained through open communications and exchanges with their loved ones [19]. Despite the longing to rekindle family bonds at the end of life, it was found that Asian patients and their families are often reluctant to engage in dignity-enhancing discourses as they feel constrained by unresolved family conflicts, and most pertinently, the cultural taboos of death. This has largely resulted in pragmatic communication and support that focus primarily on physical care with little opportunities for emotional connections.

Family, rather than the individual, is seen as the basic unit of life in Asian cultures. Hence, in order to help older terminally ill patients and their families reduce suffering and achieve a sense of hope and meaning when death draws near, family must become the driving principle that guides dignity-enhancing interventions in the Asian context [20]. Yet, despite increasing demands for holistic palliative care services in Asia with widespread population aging in the region, no such intervention is available. Building on the clinical foundation of Dignity Therapy together with the empirical understanding of dignity-related concerns of Asian families facing terminal illness, a novel “Family Dignity Intervention” (FDI) has been developed. The design of FDI is fundamentally based on the Dignity Therapy protocol, but instead of an individual model of therapy, FDI adopts a patient-family dyad model to facilitate open dialogue between patients and their family caregivers, one that aspires to strengthen family connectedness and filial compassion via creating a supportive platform for the expression of appreciation, achieving reconciliation, fortifying family bonds, and passing on transcendental values and wisdom for establishing lasting legacies. The ultimate goal of FDI is to offer a viable dignity-enhancing intervention to promote holistic palliative care in Asia, one that addresses the psycho-socio-spiritual needs of Asian families who are not strong in their expression of intimacy and articulation of emotions in the face of mortality.

### Aim and objectives

Based on the Medical Research Council guidance for developing and evaluating complex intervention [21], the aims of the current study are to assess the feasibility, acceptability, and potential effectiveness of FDI in reducing psychosocial, emotional, spiritual, and psychophysiological distress in community-dwelling and in-patient, Asian, older terminally ill patients and their families living in Singapore. The specific objectives are to:

1. Assess the effectiveness of FDI for increasing older terminally ill patients’ sense of dignity, hope, life meaning, quality of life, promoting psychophysiological wellbeing, and reducing psychosocial distress
2. Assess the effectiveness of FDI for increasing family caregivers’ sense of hope, life meaning, quality of life, promoting psychophysiological wellbeing, and improving bereavement outcomes, as well as reducing psychosocial distress
3. Assess the feasibility and acceptability of FDI for Asian palliative care in the Singaporean context, and
4. Develop a standardized protocol for further empirical research that tests intervention effectiveness, feasibility, and acceptability of FDI in other Asian regions

## Methods/design

### Study design

This study adopts an open-label randomized controlled trial design comprising of two groups: (1) an intervention group (FDI offered in addition to standard psychological care), and (2) a control group (standard psychological care which includes emotional support and psychosocial home visits). Consenting participants including one patient and one family caregiver from one family unit (i.e., patient-family dyad) are randomly allocated to one of these two groups after baseline assessment meetings have been conducted. The Standard Protocol Items: Recommendations for Interventional Trials (SPIRIT) Checklist for the study is included as an Additional file 1.

### Randomization

Randomization is conducted by an independent statistician. Treatment allocation (FDI or control) is performed by block randomization with a fixed block size of 2. Allocation concealment is facilitated by using sequentially numbered, opaque, sealed envelopes for consecutive and eligible families. To reduce the risk of bias, the researcher opens the next envelope to ascertain which group a patient-family dyad has been allocated to after baseline measures have been collected.

### Study sites

Study participants are recruited from two major hospice service providers and their various satellite centers across Singapore. First, Dover Park Hospice (DPH) is a secular, non-profit organization that offers both in-patient and homecare hospice services to terminally ill patients and their families. It is one of the largest standalone hospice in the country with over 50 in-patient beds and extensive homecare provision. Second, Hospice Care Singapore (HCA) is a registered charity that offers daycare and homecare hospice services to terminally ill patients and their families. With two day care centers and four satellite service centers stationed across the country, they provide nationwide coverage to all Singaporeans. Both DPH and HCA are funded publicly and via donations; service admissions are based on physician referrals and means-testing mechanisms as ascribed by the Singapore Ministry of Health. Both service providers house a team of palliative care specialists including physicians, nurses, social workers, and counselors to provide round-the-clock support to individuals and families facing the end of life.

### Participants

The sample comprises 126 Asian families in Singapore, which would include participants of Chinese, Malay, Indian and other Eurasian ethnicities [22]. Each family includes a patient-family dyad: (1) one older terminally ill patient and (2) one identified family member who the patient considers to be their primary or trusted caregiver. Participants are recruited through the in-patient, daycare, and homecare hospice service units of DPH and HCA. Inclusion criteria include: patients aged 60 years or above, diagnosed with a terminal illness, a life expectancy of less than 6 months, living in the community and receiving hospice-provided homecare or daycare, or residing in a long-term-care or hospice facility and receiving hospice-provided palliative care. One identified family member, who the patient considers to be their primary caregiver, is also recruited. Patients and family caregivers are screened for spiritual or psychological distress, or loss of dignity; however, these are assessed during baseline assessments to explore their potential moderating effects on the impact of FDI. Exclusion criteria include patients and family caregivers who are deemed too ill to participate, unable to provide informed consent, suffer from major cognitive disabilities, or are unable to understand and communicate in English, Mandarin, or Cantonese.

### Sample size calculation

Allowing for an attrition rate of 40% at follow-up (a large estimate due to end-of-life context and the potentiality of patients dying before completion of intervention), a sample of 126 families (*N* = 252; 126 patients and 126 family caregivers) gives us 80% power to detect an effective size of 0.6 (common medium effect size estimate for psychotherapies) [23] between the intervention and control group at the (two-tailed test) 5% level of significance.

### Intervention group

FDI is delivered by two research associates with a Master’s degrees in counseling who have received Dignity Therapy training conducted by Chochinov and his team. Training includes the theoretical foundation of dignity, an overview of the dignity therapy techniques, experiential role-play, and editing of a generativity document. The two research associates have also received training on the Asian dignity model by Ho and his team, which included the empirical understanding of dignity from the perspectives of Asian families facing end of life, basic family therapy, and meaning-oriented interview techniques, as well as experiential role-play.

Table 1 provides a summary of the intervention procedures of FDI. During the first baseline assessment meeting, a brief framing session is conducted by the FDI therapist with patient-family dyads assigned to the intervention group so as to gauge the focus of therapy. Two sets of question frameworks, one for patients and one for caregivers, are shared with the dyads to provide them with the opportunity to reflect on, and think about, their responses (see Table 2). The questions focus on eliciting patients’ life experiences in relation to their families, while caregivers’ responses are used to enrich patients’ narratives. The framing session and the question frameworks provide a flexible guide for the FDI therapist to shape the intervention interview, based on the dyads’ interest.

**Table 1:** Session content of Family Dignity Intervention (FDI)

Session	Content
1. Brief framing session	• FDI question framework provided to patient-family dyad for reflection • Therapist gauges the focus of therapy from the standpoint of the patient with input by the family caregiver in preparation for the intervention interview
2. Intervention interview session	• Recorded interview with patient-family dyad using the FDI question framework, which focuses on the patient’s life experiences in the family context, with caregiver’s responses enriching patient’s narrative • Therapist follows dyad’s cues, helps them structure and organize their thoughts, connects sequence of events, facilitates disclosure of cherished memories, and encourages expression of appreciation and reconciliation
3. Transcript review session	• Intervention interview is quickly transcribed verbatim and shaped into a coherent narrative by the therapist using a formatted editing process • Therapist meets with patient-family dyad to review the edited transcript, ensures it conveys the dyad’s overall message, and finalizes editorial revisions
4. Family sharing session	• The final transcript becomes a “legacy” document of the patient-family dyad • Therapist organizes a family meeting with the dyad and their invited loved ones to an open reading of the legacy document at a private location

**Table 2:** Question framework of Family Dignity Intervention

Questions for patients
1. Tell me a little about your life history; what are some of the most important and memorable times? When did you feel most alive?
2. How has your relationship with your loved one influenced your life?
3. What are some things you want your loved one to know about you, or to remember about you?
4. What do you think are your most important and meaningful accomplishments in life (family, career, community)?
5. What do you think your loved one is most proud of you for, or appreciates about you?
6. What do you appreciate most about your loved one?
7. Are there particular things that you want to thank your loved one for?
8. Are there particular things that you would like to ask forgiveness for, or offer forgiveness for?
9. What teachings, advice, or words of guidance do you want to pass on to your loved one?
10. What are your hopes and dreams for the future, for yourself, your loved one and your family?
11. In creating this permanent record, are there other things that you would like to include?
12. Before the session ends, are there things that you would like to take time to say again?
Questions for family caregivers
1. Tell me a little about your life history with your loved one; what are some of the most important and memorable times you had together? When did you feel most alive with your loved one?
2. How has your relationship with your loved one influenced your life?
3. What are some things you want your loved one to know about you, or to remember about you?
4. What do you think are your loved one’s most important and meaningful accomplishments in life (family, career, community)?
5. What do you appreciate most about your loved one?
6. What do you think your loved one is most proud of you for, or appreciates about you?
7. Are there particular things that you want to thank your loved one for?
8. Are there particular things that you like to ask forgiveness for, or offer forgiveness for?
9. What teachings, advice, or words of guidance have you received from your loved one, and would like to pass on to other family members?
10. What are your hopes and dreams for future for your loved one, yourself and your family?
11. In creating this permanent record, are there other things that you would like to include?
12. Before the session ends, are there things that you would like to take time to say again?

The intervention interview session takes place 2 to 3 days after the baseline meeting. During the interview using the question frameworks, the FDI therapist follows the dyads’ cues, helping them to structure and organize their thoughts. For example, the FDI therapist may ask questions about time sequences, how events are related to each other, facilitate the disclosure of cherished memories, and encourage the expression of appreciation and reconciliation. The intervention interview lasts between 60 and 90 min; it is audio-recorded, quickly transcribed verbatim then shaped into a coherent narrative using a formatted editing process. This includes clarification, chronological corrections, tagging and editing any content that might inflict significant harm on family (after discussion with the dyads), and finding a suitable ending for the document which is appropriate to the dyads’ overall message. A transcript review session is arranged 2 to 3 days later for the FDI therapist to check the edited transcript with the dyads, who are invited to make any editorial suggestions and revisions. Once the transcript is finalized, it will become a “legacy” document of the dyads, and a final family sharing session is arranged for the dyads to share and read this document to each other and their loved ones. The entire FDI, from the brief framing session, to the intervention interview session, the transcript review session, and the final family sharing session, is usually completed within 2 weeks.

Since the condition of a terminally ill patient fluctuates rapidly, the timing of contacts and meetings can be relaxed and rescheduled. If the patients’ condition deteriorates, meetings are rescheduled up to three times before sensitively withdrawing them from the study. The FDI therapist makes detailed notes of each intervention sessions and documents any deviations from the FDI protocol. One in two intervention transcripts is randomly selected for review by the principle investigator and one co-investigator for data monitoring, quality and safety assurance. These mechanisms serve as an audit trail as well as the foundation of the acceptability and feasibility study.

### Control group

Patient-family dyads in the control group receive three psychosocial home visits (four for caregivers) to be rendered by a designated research team member. Each visit comprises a research assessment interview with basic emotional support. Completing the assessment interviews provides participants with the opportunity to share their feelings and emotions along their illness trajectory. The extent to which they feel that sharing is therapeutic is explored in the interviews.

### Procedures

**Fig. 1:**
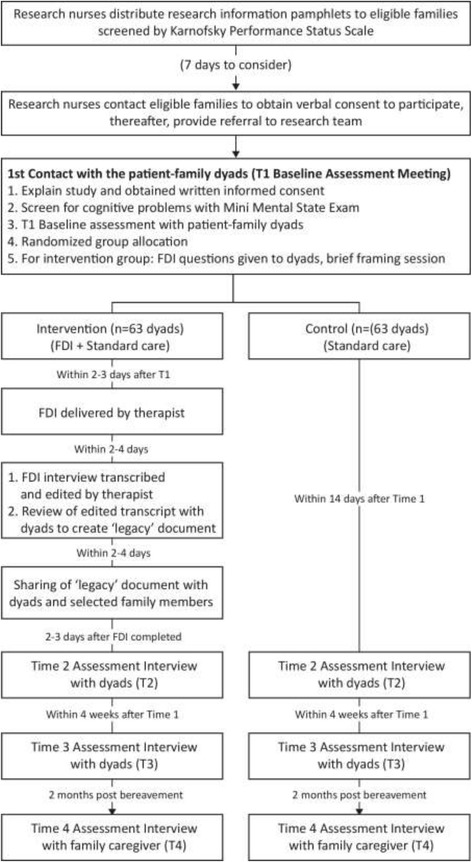
Flow diagram of recruitment and study conduct

The recruitment and follow-up procedures are shown in Fig. 1. Appointed research nurses of DPH and HCA are asked to distribute research information pamphlets to all patients and family caregivers eligible for the study, based on their clinical assessments of patients using the Karnofsky Performance Status Scale (KPSS) [24]; patients who received a score of 30 or below are considered too ill and ineligible to participate. At least 1 week is given for full consideration to participate in the study, after which, the research nurses contact each eligible family to seek their verbal consent for study participation. Once verbal consent is obtained, a simple Information Sheet containing only the names and telephone numbers of the patient-family dyad as well as referring nurses are forwarded to an appointed member of the research team. The responsible researcher then contacts the patient-family dyad via telephone to organize a convenient time for the first baseline assessment meeting. All personal information pertaining to potential participants is kept confidential and only the responsible researchers have access to such information. Information of the potential participants who refused to take part in the study upon telephone contact are destroyed immediately.

The first meeting is attended by the appointed FDI therapist, as well as a responsible researcher who (1) explains the study to the patient-family dyad, (2) answers any questions that they may have about their participation, and (3) checks that they have fully understood the implications of the study before obtaining written consent. The consent form for this study is included as an Additional file 2. As a final check, the researcher screens patients using the Mini Mental State Examination (MMSE) [25] to assess cognitive functioning, and those who score below 18 are considered unfit to participate. In such cases, patient-family dyads are excluded; this is done sensitively, whereby the researcher and the FDI therapist spend some time chatting with the patient-family dyads about neutral topics before ending the meeting. This approach has worked successfully in previous studies of a similar nature [26].

For patient-family dyads who are deemed fit to participate after the final check, the researcher conducts a baseline assessment with patients and caregivers individually, then opens the next envelope in sequence to ascertain group allocation. Dyads assigned to the control group are reminded that they will receive three psychosocial visits (four for caregivers) from the research team, through which they will have the opportunity to share their feelings along their illness trajectory. Dyads assigned to the intervention group are given the FDI framework questions, followed by a brief framing session conducted by the FDI therapist. A time is then arranged for the intervention interview session within the next 2 to 3 days.

After the intervention interview is completed and the recorded transcript has been edited and finalized with the patient-family dyad via a transcript review session, the FDI therapist sets up a time for a final family sharing session in which the “legacy” document is shared with selected members of the patients’ family. A hard copy of the legacy document is also given to the patient-family dyad for safekeeping.

### Outcome measures

**Fig. 2:**
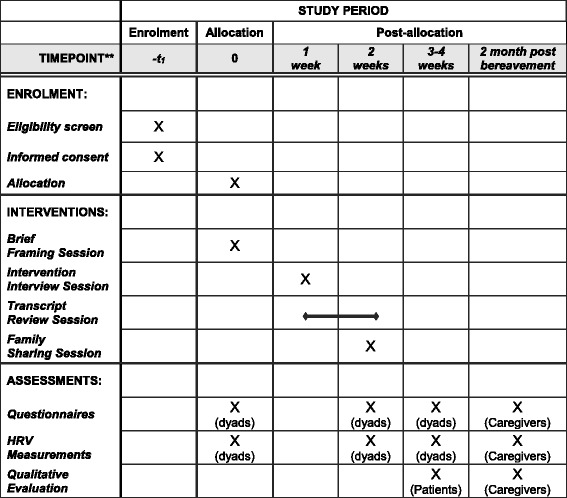
Schedule of enrollment, intervention, and assessment

Both quantitative outcomes (subjective and objective measures) as well as qualitative outcomes are assessed with patients and family caregivers through face-to-face interviews. For patient-family dyads assigned to the intervention group, these are assessed at four time points (see Fig. 2 for the corresponding Standard Protocol Items: Recommendations for Interventional Trials (SPIRIT) Figure): during the initial baseline assessment meeting (T1), approximately 14 days after baseline (i.e., 2 to 3 days after the legacy documents have been shared in the final family sharing session) (T2), 4 weeks after baseline (i.e., 2 weeks after intervention completion) (T3), and during an exit interview with family caregivers after the patients’ death at 2 months post bereavement (T4) for assessing grief outcomes. Equivalent assessments are conducted with patient-family dyads assigned to the control group at the same four time points: baseline assessment meeting (T1), approximately 14 days (T2) and 4 weeks (T3) after baseline, as well as an exit interview with family caregivers at 2 months post bereavement [T4]. All quantitative measures have been validated with older populations, and demonstrated strong internal consistency, test-retest reliability, construct and concurrent validity. Some measures have been designed specifically for end-of-life care research while others have frequently been adopted in palliative care settings; all measures are used widely in Asian contexts. Qualitative interview schedules have been developed for this study.

### Subjective outcome measures for patients

The primary outcome is patients’ sense of dignity (potential effectiveness of FDI). This is assessed at baseline (T1), 14 days (T2), and 4 weeks’ follow-up (T3), using the Patient Dignity Inventory [27]. This 25-item measure evolved directly from the dignity model and is comprised of questions relevant to the physical, psychosocial, and spiritual domain of end-of-life concern or distress. Secondary outcomes are assessed using: Patient Health Questionnaire-9 [28] (psychological distress); a modified version of the Cancer Coherence Scale [29] (meaning in life); World Health Organization Quality of Life Scale-8 [30] (quality of life); FACIT Spiritual Well-being Scale [31] (spirituality); Herth Hope Index [32] (hopefulness); and a modified version of the Inventory of Social Support [33] (perceived support).

### Subjective outcome measures for caregivers

The primary outcome is caregivers’ psychological distress (potential effectiveness of FDI). This is assessed at baseline (T1), 14-day (T2), and 4-week (T3) follow-up, as well as at 2 months (T4) post bereavement by the Patient Health Questionnaire [28]. Secondary outcomes are assessed using: a modified version of the Cancer Coherence Scale [29]; World Health Organization Quality of Life Scale-8 [30]; FACIT Spiritual Well-being Scale [31]; Herth Hope Index [32]; and a modified version of the Inventory of Social Support [33]. Moreover, the Brief Grief Questionnaire [34] and the Core Bereavement Items [35] is used to assess grief and bereavement outcomes at 2 months post bereavement.

### Objective outcome measures for patient-family dyads

Heart rate variability (HRV), a reliable biomarker that reflects an individual’s cardiovascular regulation, is used to assess participants’ psychophysiological wellbeing. Specifically, HRV scores are measured as the duration of the inter-heartbeat interval, which mirrors balance in the autonomic nervous system [36]. Higher HRV scores indicate stronger responsiveness of the autonomic nervous system in changing physiological arousal levels to suit situational demands and life stressors, and serve as a potential indicator of wellbeing and longevity; whereas lower HRV scores indicate poor responsiveness of the autonomic nervous system, which has been associated with psychological conditions like anxiety and depression [37]. ithlete [38], a small non-invasive portable HRV measurement device, together with a Smart tablet installed with the ithlete HRV app, is used for this assessment. Concisely, an infrared pulse plethysmography finger sensor is attached to participants’ index finger while following a series of brief breathing instructions on the screen of the Smart tablet, and during this 1–2-min exercises, data of their psychophysiological performance are collected. As the procedure requires participants to be in a restful state so as to minimize the artefacts in heart rate data, HRV assessment is conducted as the end of each face-to-face assessment interview.

### Demographic measures

To determine whether medical and demographic variables are potential confounding factors that affect primary and secondary outcome variables, clinical information (i.e., disease types, time since diagnosis, and stages of disease) is collected from patients at baseline assessment. Demographic data (i.e., age, gender, marital status, family composition, living arrangements, education, ethnicity, and religion) are also collected during baseline assessment from both patients and family caregivers. Information on the counseling service received is also sought.

### Qualitative outcome evaluation

Together with the quantitative assessment, a series of simple, open-ended, evaluative questions are used to seek participants’ insights into their evolving psycho-social-spiritual needs along the illness trajectories, the impact of FDI on themselves and their families, as well as their views on taking part in this study. All participants assigned to the intervention group who have completed FDI are interviewed by an assigned researcher at the third (T3) (for patients) and fourth (T4) (for family caregivers) assessment time points; all interviews are recorded and transcribed for analysis. With participants’ consent, qualitative data analysis is also carried out using the FDI transcripts to identify insights into concerns which might impact intervention effectiveness, as well as, narratives and stories of dignity, hope, and meaning at life’s end.

### Acceptability and feasibility assessment

To assess the implementation and delivery of FDI in community settings, time needed to organize and conduct the intervention sessions, transcribe and edit narratives; deviations from the intervention protocol, uncompleted interventions and their reasons; and the FDI therapists’ perceptions of competence as a result of training, are recorded. FDI therapists also record their experiences of delivering the intervention, their observations of patients’ and caregivers’ responses during and after the intervention, as well as any difficult or unusual cases.

### Data analysis

#### Quantitative data

All quantitative data are entered, stored, and analyzed using SPSS statistical analysis software. Between- and within-participant comparisons of outcomes are conducted and the appropriate effect size estimates reported. The intervention group and the control group are compared on the main outcomes (sense of dignity for patients; psychological distress for caregivers) and secondary outcomes (psychological distress, meaning in life, quality of life, spirituality, hopefulness, perceived support, and psychophysiological wellbeing for patients; meaning in life, quality of life, spirituality, hopefulness, perceived support, psychophysiological wellbeing, and bereavement outcome for caregivers). Comparisons are done at T2 and T3 follow-ups with baseline for both patient groups on these measures, as well as at T2, T3, and T4 follow-ups with baseline for both caregiver groups. To characterize and predict changes in outcome variables, multilevel analyses are conducted. The intervention and control groups are also compared on demographic characteristics and baseline measures. If necessary, these are controlled in the analyses. Exploratory analyses on the influence of cultural, ethnical, and religious factors on main outcomes and secondary outcomes are also carried out. Finally, recruitment rates, as well as comparisons of dropout rates and missing data in the two groups are reported.

#### Qualitative data

The Framework method of analysis is used [39]. Analysis is both deductive (from pre-set aims and objectives) and inductive (arising from participants’ view). This method tends to be more structured than some other methods of qualitative analysis and the process is more explicit and more informed by a priori questions. It is designed so that it can be easily understood and assessed by people other than the analysts such as funding bodies, policy-makers, and participants. Throughout the analytical process, strategies to maximize credibility, criticality, and authenticity are applied. The QSR NVIVO software package is used to manage the data.

## Discussion

In Asia and particularly in Singapore, Hong Kong, China, Japan, Korea, and their southeastern neighbors, demand for palliative care has surged over the past decade and will continue to rise in the future under the context of population aging [40]. Regional and local governments have honorably aspired to enhance the provision of holistic palliative care to patients and families facing chronic and terminal illnesses. However, most palliative interventions still focus predominately on pain and symptom management without addressing psycho-socio-spiritual concerns. To date, there is no available palliative care intervention for dignity enhancement in Singapore nor in Asia [20]. To address the critical gaps in the provision of holistic palliative care services for patients and families facing terminal illness, death, and bereavement, the research team designed a randomized controlled trial that (1) integrates empirical knowledge on end-of-life dignity-related needs and concerns from both Western and Asian contexts, as well as well-researched therapeutic model for dignity enhancement, to form a culture-specific FDI for Asian palliative care; (2) tests the effectiveness of this novel intervention model (FDI) on enhancing the psycho-socio-spiritual wellbeing of terminally ill patients and their families receiving palliative care in Singapore; and (3) establishes a standardized intervention research protocol for further testing of FDI in other societies across Asia that have similar cultural and ethnic contexts.

The patient-family dyad intervention model of the FDI supports the unique needs of Asian families who are not accustomed to, or comfortable with, the expression of emotions and affections even during times of loss and separation [16, 17]. The lack of open communication between patients and their family members prevents the opportunities for reconciliation, resolving “unfinished business,” as well as the expression of love and appreciation, all of which are pivotal processes for minimizing the existential pain of mortality. Creating a safe and caring platform for patients and families to engage in such intimate discourses could greatly elevate the experience of dying, while reducing the psychosocial and spiritual pain that is often deemed to be of greater concern to older terminally ill patients than physical pain [18]. Authentic dialogues on life and death could further cultivate empathetic understanding between patients and their family caregivers, fostering the sense of filial compassion that imperatively fuels and sustains devotion to family caregiving at life’s end [19]. This is of particular importance in Asia given that many Asian governments have established their policies for older people on notions of aging in place and dying at home [40], both of which accentuate the significant role that the family plays in end-of-life caregiving. FDI could be a vital mediator to support such policy agendas.

The FDI dyad intervention model could further promote family centered care, a highly valued provisional practice of palliative services in Asia [20], one that transcends conventional person-centered care in Western societies. In fact, FDI could serve as the starting point to engage patients and their families with professional caregivers in honest and constructive communications, those that foster not only the expression of emotions, but also encourage partnerships in familial and professional caregiving, as well as cultivating informed participation in the decision-making of end-of-life care treatments and options. Repeated research has shown that joint participation in the governance of mortality between professional caregivers and family caregivers is indispensable in alleviating dying patients’ anxiety and depression, and minimizing unnecessary and futile treatments that may bring more harm than good, in addition to reducing clinician-patient-family conflicts that are often found amidst the unmanaged chaos and confusions brought on by death and dying [41]. This is also in line with national policies on advance care planning (ACP) that have emerged across Asia in recent years, whereby governments are beginning to recognize the importance of promoting civil responsibility in preparation for death and informed-care decision-making at the end of life among members of the public. In Singapore, for example, since 2011 the Ministry of Health has invested millions of dollars in developing a national ACP program [42]. But after years of effort and resources spent on training health and social care workers to engage patients and families in ACP conversations, less than 10,000 or so ACP conversations have been completed in the past 6 years among a population of 5.5 million with nearly 20,000 deaths per year [43]. This clearly reflects an imminent need to develop better strategies to engage patients and families in ACP discussions, and FDI can certainly pave the way for such dialogues to take place via elevating the emotional readiness of patients and families to talk openly and candidly about mortality.

In terms of the intervention protocol developed by this study, its brief and systematic design will allow clinicians to implement and assess the feasibility of adopting FDI in different clinical and community care settings. The transparent and meticulous evaluation design proposed will also enable researchers to test its effectiveness in promoting dignity and holistic wellbeing among different population age groups facing terminal illness, not limited to older adults. The inclusion of an exit assessment with family caregivers during bereavement contributes to the longitudinal database for studying grief outcomes, and can further inform the development of other anticipatory grief therapy and counseling modalities. Finally, the inclusion of heart rate variability to measure the psychophysiological wellbeing of study participants provides another innovative and objective method to assess the effectiveness of a psycho-socio-spiritual intervention for palliative care.

### Limitations and recommendations

Despite the study’s strengths, some limitations do exist. First of which is the limitation posed by involving only two end-of-life care providers in the study, both of which are established in-patient and home-care hospices that are highly proficient in caring for the dying and the bereaved. This may impact the outcome of FDI given the high-quality standard care rendered to patients and families; thus, future research can expand study sites to include hospitals and nursing homes to assess the acceptability and effectiveness of FDI in primary, acute, and long-term-care settings. Second, only patients who attained a score of 30 and above from the KPSS are deemed fit to participate in the study. This screening criterion may exclude individuals who are very sick but may still have the capacity to engage in, and benefit from, the FDI; hence, future studies and clinical practices may consider lowering this criterion to work with patients with sufficient functional capacity to engage in a therapeutic narrative dialogue. Third, in consideration that terminally ill patients’ HRV may be affected by their physical states as well as their prescribed medications, future research may consider collecting relevant biometric data, such as patients’ weight, height, and Body Mass Index as well as the type of medications prescribed, for cross-comparison and to ensure the validity of HRV measurements [44]. Finally, due to the limited language ability of the research team, FDI can only be conducted with English-, Mandarin- and Cantonese-speaking participants. While Singapore is an ethnically diverse nation with many residents of various cultural backgrounds and language abilities [22], it is unreasonable to presume that the findings generated from this study can be applied to the majority of Asian populations. Therefore, future research needs to expand the delivery languages of FDI, as well as to assess its acceptability and effectiveness among the many different ethnic groups in greater Asia.

With the aforesaid, this first-of-its-kind study develops and tests an evidence-based and family driven psycho-socio-spiritual intervention for enhancing dignity and wellbeing among patients and families facing mortality. It addresses a critical gap in the provision of holistic palliative care services. The expected outcomes should generate new knowledge contributing to advancement in both clinical theories and practices in palliative care for Singapore and its neighboring regions, while serving to inform similar developments in other Asian communities.

### Trial status

The RCT is currently in month 10 of 36 planned months of recruitment and data collection.

## Additional files

Additional file 1: Standard Protocol Items: recommendations for Interventional Trials (SPIRIT) 2013 Checklist: recommended items to address in a clinical trial protocol and related documents. (DOC 125 kb)

Additional file 2: Participant Informed Consent Form. (DOCX 78 kb)

## Abbreviations

ACP: Advance care planning
DPH: Dover Park Hospice Singapore
FDI: Family Dignity Intervention
HCA: HCA Hospice Care Singapore
HRV: Heart rate variability
KPSS: Karnofsky Performance Status Scale
MMSE: Mini Mental State Exam
