# Supplementary material for: A novel Family Dignity Intervention (FDI) for enhancing and informing holistic palliative care in Asia: study protocol for a randomized controlled trial
Source: Trials. 2017 Dec 4;18:587. doi: 10.1186/s13063-017-2325-5 (PMC5715529; doi:10.1186/s13063-017-2325-5)
Supplement: Supplementary file 2 — Participant Informed Consent Form. (DOCX 78 kb) [file 13063_2017_2325_MOESM2_ESM.docx]

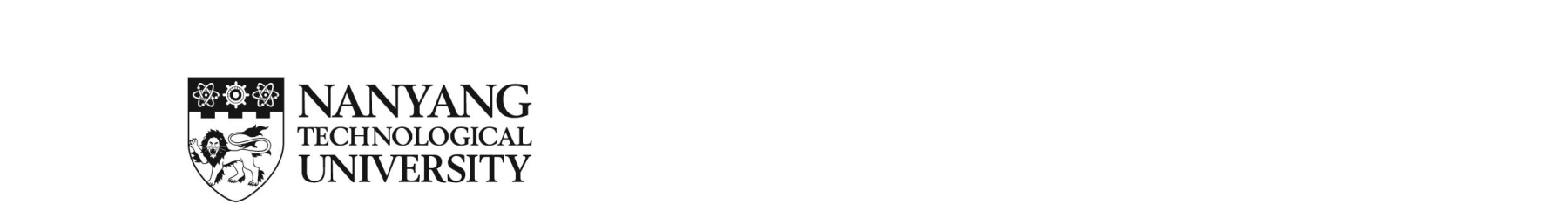
A Multicentre Randomized Controlled Trial of a Novel **Family Dignity Intervention (FDI)**
for Asian Palliative Care

**Patient’s Informed Consent Form**

You are invited to participate in a research study conducted by Dr. Andy H. Y. Ho, Assistant Professor of Psychology, School of Social Sciences, Nanyang Technological University, and in collaboration with Dover Park Hospice and HCA Hospice Care, Singapore.

**Purpose of the Study**

The current study aims to develop and test a novel “Family Dignity Intervention” (FDI) for Asian patients and family caregivers receiving palliative care, with an aspiration to create a supportive and constructive platform that fosters the expressions of appreciation, achieving reconciliation, fortifying family bonds, and passing on wisdom and values across generations.

**Procedures**

Your participation in the FDI study will span across 4 sessions within a 1-month period. Working with an experienced counsellor, you will be provided with opportunities to review, create and share a legacy document through exploring meaningful life experiences, important relationships, and wisdoms of dignified living with your family. You will also be required to complete 3 sets of standardized questionnaires together with a brief psychophysiological test (via a breathing-based non-invasive portable device) at the start, end and 2 weeks after completing the FDI via face-to-face interviews. Each FDI session inclusive of questionnaire completion will last between 60-90 minutes, and the intervention components of the FDI will be audio-recorded for analysis.

**Potential Risk**

There is minimal risk for engaging in activities of life review and life sharing. An experienced counsellor will be available on-site to offer support in the event that some aspects of the study causes you distress or discomfort. If you need further support, a referral can be made to the psycho-social team of your healthcare provider, and they will be able to provide you with follow-up assistance and consultations.

**Potential Benefits**

Your participation in the FDI study will bring about opportunities for increasing patient and caregiver’s sense of dignity, strengthening family bonds and creating shared memories and wisdom to be gifted to future generations. We hope that you will experience satisfaction from being a part of this study, while helping us to develop enhanced and meaningful family-orientated interventions for Asian patients and families receiving palliative care.

**Compensation**

You will receive a $30 cash voucher for each completed assessment, totaling $90, as a token of our appreciation of your time and participation.

**Confidentiality**

All personal information and responses provided by you in the study will be treated as strictly confidential. Study data and audio records of the FDI sessions will be stored securely and will be made available only to persons conducting the study unless participants specifically give permission in writing to do otherwise. In follow-up reports and publications, your identifying information will be obscured or omitted.

**Participation and Withdrawal**

Your participation is voluntary. This means that you can choose to stop at any time without negative consequences.

**Questions and Concerns**

If you have any questions about this study, please contact Dr. Andy H. Y. Ho at Nanyang Technological University [14 Nanyang Drive, HSS-04-03, Singapore 637332; Telephone: 6316-8943; Email: andyhyho@ntu.edu.sg]. If you have questions regarding your rights as a research participant, please contact the Institutional Review Board of Nanyang Technological University [Telephone: 6592-2495].

**SIGNATURE**

I _________________________________ (Name of Participant) understand the procedures described above and agree to participate in this study.

I agree / do not agree to the audio-recoding during the procedure.

__________________________________ ­­­­­­­­­­­­­­­­­­­­­­­­­­­­­­­­

Signature of Participant Date

_____________________________________ _________________________________

Name & Signature of Researcher/Interviewer Date

Research Ethnics Committee Approval Expiration date: [30/01/2020]

# **Appendix B: Informed consent for family caregivers**


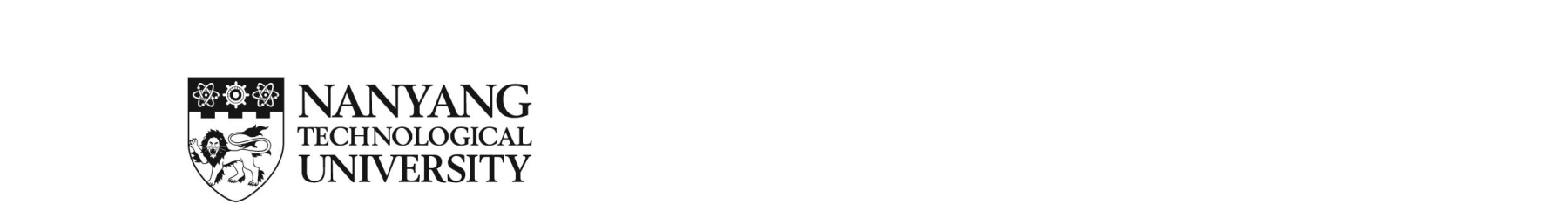
A Multicentre Randomized Controlled Trial of a Novel **Family Dignity Intervention (FDI)**
for Asian Palliative Care

**Caregiver’s Informed Consent Form**

You are invited to participate in a research study conducted by Dr. Andy H. Y. Ho, Assistant Professor of Psychology, School of Social Sciences, Nanyang Technological University, and in collaboration with Dover Park Hospice and HCA Hospice Care, Singapore.

**Purpose of the Study**

The current study aims to develop and test a novel “Family Dignity Intervention” (FDI) for Asian patients and family caregivers receiving palliative care, with an aspiration to create a supportive and constructive platform that fosters the expressions of appreciation, achieving reconciliation, fortifying family bonds, and passing on wisdom and values across generations.

**Procedures**

Your participation in the FDI study will span across 4 sessions within a 1-month period, with an additional exit interview session. Working with an experienced counsellor, you will be provided with opportunities to review, create and share a legacy document of your loved one through exploring meaningful life experiences, important relationships, and wisdom of dignified living that define his or her life. You will also be required to complete 4 sets of standardized questionnaires together with a brief psychophysiological test (via a breathing-based non-invasive portable device) at the start, end, 2 weeks after completing the FDI, and at the final exit interview via self-administration or face-to-face interviews. Each FDI session inclusive of questionnaire completion will last between 60-90 minutes, and the intervention components of the FDI will be audio-recorded for analysis.

**Potential Risk**

There is minimal risk for engaging in activities of life review and life sharing. An experienced counsellor will be available on-site to offer support in the event that some aspects of the study causes you distress or discomfort. If you need further support, a referral can be made to the psycho-social team of your healthcare provider, and they will be able to provide you with follow-up assistance and consultations.

**Potential Benefits**

Your participation in the FDI study will bring about opportunities for increasing patient and caregiver’s sense of dignity, strengthening family bonds and creating shared memories and wisdom to be gifted to future generations. We hope that you will experience satisfaction from being a part of this study, while helping us to develop enhanced and meaningful family-orientated interventions for Asian patients and families receiving palliative care.

**Compensation**

You will receive a $30 cash voucher for each completed assessment, totaling $120, as a token of our appreciation of your time and participation.

**Confidentiality**

All personal information and responses provided by you in the study will be treated as strictly confidential. Study data and audio records of the FDI sessions will be stored securely and will be made available only to persons conducting the study unless participants specifically give permission in writing to do otherwise. In follow-up reports and publications, your identifying information will be obscured or omitted.

**Participation and Withdrawal**

Your participation is voluntary. This means that you can choose to stop at any time without negative consequences.

**Questions and Concerns**

If you have any questions about this study, please contact Dr. Andy H. Y. Ho at Nanyang Technological University [14 Nanyang Drive, HSS-04-03, Singapore 637332; Telephone: 6316-8943; Email: andyhyho@ntu.edu.sg]. If you have questions regarding your rights as a research participant, please contact the Institutional Review Board of Nanyang Technological University [Telephone: 6592-2495].

**SIGNATURE**

I _________________________________ (Name of Participant) understand the procedures described above and agree to participate in this study.

I agree / do not agree to the audio-recoding during the procedure.

___________________________________ _________________________________

Signature of Participant Date

_____________________________________ _________________________________

Name & Signature of Researcher/Interviewer Date

Research Ethnics Committee Approval Expiration date: 30/01/2020
